# Microsatellite Loci Analysis Reveals Post-bottleneck Recovery of Genetic Diversity in the Tibetan Antelope

**DOI:** 10.1038/srep35501

**Published:** 2016-10-14

**Authors:** Yurong Du, Xiaoyan Zou, Yongtao Xu, Xinyi Guo, Shuang Li, Xuze Zhang, Mengyu Su, Jianbin Ma, Songchang Guo

**Affiliations:** 1 School of Life and Geography Science, Qinghai Normal University, Xining 810008, China; 2 Key Laboratory of Evolution and Adaptation of Plateau Biota, Northwest Institute of Plateau Biology, Chinese Academy of Sciences, Xining 810007, China; 3 School of Life Science, Sichuan University, Chengdu 610064, China; 4 School of Chemistry and Chemical Engineering, Qinghai University for Nationalities, Xining 810001, China

## Abstract

The Tibetan antelope (chiru, *Pantholops hodgsoni*) is one of the most endangered mammals native to the Qinghai-Tibetan Plateau. The population size has rapidly declined over the last century due to illegal hunting and habitat damage. In the past 10 years, the population has reportedly been expanding due to conservation efforts. Several lines of evidence suggest that the Tibetan antelope has undergone a demographic bottleneck. However, the consequences of the bottleneck on genetic diversity and the post-bottleneck genetic recovery remain unknown. In this study, we investigate the genetic variation of 15 microsatellite loci from two Tibetan antelope populations sampled in 2003 (Pop2003) and 2013 (Pop2013). A higher level of genetic diversity (*NA*, 13.286; *He*, 0.840; *PIC*, 0.813; *I*, 2.114) was detected in Pop2013, compared to Pop2003 (*NA*, 12.929; *He*, 0.818; *PIC*, 0.789; *I*, 2.033). We observe that despite passing through the bottleneck, the Tibetan antelope retains high levels of genetic diversity. Furthermore, our results show significant or near significant increases in genetic diversity (*He*, *PIC* and *I*) in Pop2013 compared with Pop2003, which suggests that protection efforts did not arrive too late for the Tibetan antelope.

The Tibetan antelope or chiru (*Pantholops hodgsonii*) is a member of the Bovidae family (order Artiodactyla) native to the Qinghai–Tibet Plateau (QTP). It is distributed primarily throughout central Tibet, Qinghai, Xinjiang and west Sichuan in China, occupying a total area of approximately 880,000 km^2^ at an altitude of 3700 to 5500 m^1^. The Tibetan antelope was widely distributed over the QTP in the 20^th^ century, with a population size ranging from 500,000 to 1,000,000 individuals during peak years^2^. However, this species has suffered a severe demographic bottleneck since the 1950s. The estimation of Tibetan antelope population size reached the minimum number of 50,000 individuals in 2003^3^. Such a drastic reduction is primarily considered to be the result of human activity such as poaching and overgrazing. Accordingly, the Tibetan antelope is listed as “Endangered” on the IUCN Red List (http://www.iucnredlist.org/details/15967/0) and as a Category I species under the Wild Animal Protection Law of China. To protect the Tibetan antelope and restore the population, the Chinese government has set up seven Nature Reserves since 1993^4^ and has taken measures such as anti-poaching, building wildlife passageways, and strengthening publicity. In 2011, the number of Tibetan antelope was estimated to have recovered up to 200,000 (Antelope Specialist Group 2011, See ref. 3).

Nevertheless, Tibetan antelope populations are still suffering from pressure from poaching, the development of society by man, and the worsening environment as a result of global warming and grassland degradation caused by human activities. Genetic variant analysis based on SSR and mtDNA markers in the giant panda^5^, grey shrike^6^ and Sichuan snub-nosed monkey^7^ suggest that declines in population lead to the rapid loss of genetic diversity and, thus, the danger of population extinction. Although the Tibetan antelope population continues to grow, it is unclear whether the genetic diversity of this species is increasing over time. Furthermore, the recovery of endangered species from severe population bottlenecks now frequently involves human intervention, but the genetic consequences of intervention strategies remain unknown. Herein, we address this gap in knowledge by using 15 microsatellite loci to investigate temporal changes in the genetic diversity of Tibetan antelope during an 11-year period. The aim of this study was to examine the trends in this change and to what extent the populations have been restored at the genomic level. Our results provide guidance for future conservation and management strategies.

## Materials and Methods

### Materials

Skin tissue samples were obtained from Qinghai Forest Bureau in 2003, which confiscated Tibetan antelopes that were poached in the Hoh Xil National Nature Reserves (hereafter referred to as Pop2003, n = 47). Placental tissues were collected from the Zhuonaihu Lake area in Hoh Xil National Nature Reserve in 2013 (hereafter referred to as Pop2013, n = 47). All samples were washed three times with deionized water, sucked with 95% ethanol and stored at −80 °C. All necessary approvals for collection and experimentation were acquired for the described field study from the Forestry Department of Qinghai Province, China. All procedures were in accordance with the guidelines of the regulations of experiments on animals and were approved by the China Zoological Society.

### Microsatellite loci and primers

A total of 15 polymorphic microsatellite loci were screened. Seven loci (locus P1, locus P9, locus P17, locus P24, locus P113, locus P154 and locus P160) were obtained using the FIASCO method^8^, and another 8 loci (locus P6, locus P63, locus P67, locus P73, locus P75, locus P78, locus P90 and locus P96) were searched and developed from the Tibetan antelope genome using Perl script and MISA software (http://pgrc.ipk-gatersleben.de/misa/misa.html) for extracting the microsatellite DNA sequences from genome DNA of chiru (http://www.ncbi.nlm.nih.gov/Traces/wgs/?val=AGTT01#contigs). The forward microsatellite primers were labeled by FAM and synthesized by Sangon Biotech (Shanghai). The primers specific for 15 microsatellite loci are shown in Table 1.

### DNA extraction

Genomic DNA was extracted using the standard SDS-Phenol method^9^ with minor modifications. The concentration and purity of genomic DNA were measured by Nano 2000C, and template DNA was diluted to 50 ng/μL. All loci were amplified in each of the 47 samples of Pop2003 and Pop2013. The PCR amplification was conducted in a 20-μL reaction mixture, which contained 0.1 mmol/L dNTPs, 0.2 μmol/L each of primers, 1 Unit *Taq* DNA polymerase and 1 μL DNA template. The PCR programme was set at 94 °C for 2 min, followed by 35 cycles at 94 °C for 30 s, x °C (x = the annealing temperature specified for each set of primers, (Table 1) for 30 s and 72 °C for 30 s. A final step of 10 min at 72 °C completed the programme. GeneScanTM-500 LIZTM was used as internal standard for Polymorphism detection on a ABI 3730 DNA Sequencer (Applied Biosystems, Inc.). GeneMarker V1.75 (Applied Biosystems, Inc.) was used for genotype interpretation.

### Statistical analysis of genetic diversity parameters

Original data were analyzed and manually corrected to validate the accurate peak shape and allele size using GeneMarker V1.75. Micro-Checker was used to validate the availability of genotype data^10^. The transformations for genotype data formats were conducted by Convert V1.3.1^11^ for subsequent analysis. Linkage disequilibrium (LD) test was analyzed by Genepop V4.4^12^,^13^. Number of Alleles (*NA*) and Shannon index (*I*) were calculated by Microsatellite Analyzer V4.05^14^. We calculated observed heterozygosity (*Ho*), expected heterozygosity (*He*) and analyzed the Hardy-Weinberg equilibrium (HWE) by implementing Arlequin V3.5^15^. Polymorphism information content (*PIC*) was estimated by modified PowerStats Worksheet^16^. BOTTLENECK V1.2.02^17^,^18^ was used to validate whether the population had undergone the bottleneck effect. Under a two-phase model (TPM), we constrained the model by defining 90% of mutations as conforming to a stepwise mutation model and 10% as multi-step. Furthermore, the change in Ne was estimated under the infinite allele model according to the formula *Θ* (theta) = 4Ne × Mu, where Ne is the effective population size and Mu is the microsatellite mutation ratio.

## Results

### Hardy-Weinberg equilibrium

For the 15 polymorphic loci surveyed, a total of 223 alleles were observed in both Pop2003 and Pop2013, 192 alleles were found in Pop2003, and 197 alleles were found in Pop2013. Overall microsatellite variability was high (8–19 alleles per locus) in Pop2003, and *NA* varied from 8 to 21 in Pop2013 (Table 2). Among all of the 15 loci, only 8 alleles at locus P6 were detected in Pop2003 or Pop2013, while the maximum number of alleles was found at locus P113 (19 and 21 alleles in Pop2003 and Pop2013, respectively). At each locus, the frequencies differed significantly among alleles. The lowest allele frequency was 1, accounting for 1.1% of all alleles, but the highest frequency was 46, accounting for 48.9% of all alleles. HWE test showed significant deviations from HWE at locus P17, locus P67, locus P73 and locus P154 (*P* < 0.001). However, significant deviations from HWE were confirmed only at locus P73 and locus P154 in Pop2003 and locus P17 and locus P73 in Pop2013 after Bonferroni correction (adjusted α = 0.05/15).

### Linkage disequilibrium test

At locus P73, significant LD was observed in both Pop2003 and Pop2013 (*P* < 0.05). We therefore excluded this locus in subsequent analysis. Over 14 microsatellite loci, microsatellite loci pairs P1/P9, P9/P78 and P24/P96 were in LD (*P* < 0.05). However, no LD was detected between these locus pairs after Bonferroni corrections (adjusted α = 0.05/14). In Pop2013, there was significant LD between 55 locus pairs (between locus P1 and P9, P24, P75, P78, P96, P113, P154, P160; locus P6 and P9, P75, P113, P160; locus P9 and P24, P75, P78, P90, P96, P113, P154, P160; locus P17 and P24, P75, P113; locus P24 and P63, P67, P75, P78, P96, P113, P154, P160; locus P63 and P67, P75, P96, P113, P154, P160; locus P67 and P75, P113, P154; locus P75 and P78, P96, P113, P154, P160; locus P75 and P78 and P96, P113, P160; locus P78 and P96, P113, P154, P160; locus P154 and P160). However, only 22 locus pairs were found to be in significant LD after applying Bonferroni corrections for multiple tests (Table S1). Our results suggested that these microsatellite loci were relatively independent in the Tibetan antelope.

### Genetic diversity

Microsatellite data of 14 microsatellite loci revealed abundant genetic diversity in both Pop2013 and Pop2003 (Table 1). In Pop2003, the mean number of alleles (*MNA*) was 12.92 (range 8–19), the average *Ho* was 0.832 (range 0.660–0.979), and the average *He* was 0.810 (range 0.636–0.925). In Pop2013, the average of *NA*, *Ho* and *He* across 14 loci was 13.133, 0.810 and 0.840, respectively; *NA* varied from 8 to 21, *Ho* varied from 0.556 to 0.957 and *He* varied from 0.721 to 0.920. The *PIC* at each microsatellite locus was always larger than 0.5 (range 0.561 to 0.909), a threshold value considered to be highly informative. Shannon information index ranged from 1.240 to 2.678, which also indicated a high genetic diversity.

### Bottleneck effect

The infinite allele model (IAM), TPM and stepwise mutation model (SMM) were applied when BOTTLENECK was used to test for population bottlenecks in this study. Populations exhibiting a significant heterozygosity excess would be considered to have experienced a recent genetic bottleneck. Under the TPM, the results displayed no genetic bottleneck effect in either Pop2003 or Pop2013 (Table 3). Furthermore, the Wilcoxon test, which is considered to be more reliable than the sign test and standardized differences test, showed no significant results for population bottleneck under the SMM (Pop2003: *P* = 0.923; Pop2013: *P* = 0.985). The sign test, standardized differences test and Wilcoxon test all showed a significant heterozygosity excess in either Pop2003 or Pop2013 under the IAM (*P* < 0.05). However, this was not necessarily indicative of true heterozygosity excess, as the IAM is thought to be a less appropriate model for microsatellites than the SMM^19^. These results thus point to the population resilience of the Tibetan antelope.

### Recovery of genetic variation

A tendency toward recovery of genetic diversity was observed in the Tibetan antelope. Pop2013, compared with Pop2003, exhibited higher values in *He*, *PIC*, *I* and *Θ* at 9 out of 14 loci (locus P1, P6, P9, P24, P63, P75, P90, P154, P160). In comparison with Pop2003, paired t-test demonstrated that Pop2013 had significantly higher values of average *He* (0.840 Vs 0.818, *P* < 0.05) and *PIC* (0.813 Vs 0.789, *P* < 0.05) but differences in average *MNA* (13.133 Vs 12.929, *P* > 0.05) and *Ho* (2.144 Vs 2.033, *P* > 0.05) were not found (Table 2). There was also a near significant rise (P = 0.060) in *I*, indicating genetic recovery in the Tibetan antelope. Furthermore, the average *Θ* increased from 4.581 (Pop2003) to 5.305 (Pop2013) over an 11-year period (Table 4), showing that the population size had increased significantly over approximately a decade (*P* < 0.05).

## Discussions

### Genetic diversity

We examined 15 microsatellite loci in this study to assess the genetic variation in the Tibetan antelope. Our data show rich genetic diversity in Pop2003 and Pop2013 with high values in *MNA*, *He* and *PIC*. Although the results are not directly comparable because different microsatellite loci were used, comparisons of the Tibetan antelope to other Bovidae species suggest that genetic diversity in the Tibetan antelope ranks highest (Table 5), with values of 12.929/13.286 and 0.818/0.840 for *MNA* and *He* in Pop2003 and Pop2013, respectively. Compared to the domestic yak, which is herded in the QTP and the adjacent Asian highlands with a population of more than 14 million, the Tibetan antelope exhibits higher genetic variation (Table 5). These results are in agreement with a previous genetic analysis^20^ and our previous study^21^, in which significant heterogeneity in the frequencies of mtDNA control region haplotypes was observed.

### Bottleneck signature detection

Bottleneck detection is critical for interpreting the historical demography of populations and is informative for establishing conservation strategies for endangered animals. Simulations inferred from mtDNA D-loop fragment show that the Tibetan antelope experienced a severe historical demographic decline since approximately five thousand years before present (B.P.)^21^. Although the Wilcoxon test detected no significant recent population bottleneck signature in the Tibetan antelope under the TPM and SMM via BOTTLENECK in the present study, a recent well-documented decline in the population size of the Tibetan antelope has occurred over the last century, with population size decreasing from a maximum of approximately 500,000–1,000,000 to a minimum of 50,000^2^,^3^. Heavy illegal poaching associated with a profitable fur trade could account for this severe demographic reduction. The bottleneck was not detected using the Wilcoxon test for heterozygous excess probably because the number of loci analyzed was small^17^,^18^ or due to an insufficient sample size^17^.

### Slow post-bottleneck recovery of genetic diversity

Demographic bottlenecks can result in a loss of genetic variation^22^,^23^,^24^,^25^,^26^ due to the bottleneck effect and subsequent genetic drift, as has already been observed in African elephants^27^, black-footed ferrets^28^ and Arctic foxes^29^. Rather than rapid genetic loss, the results presented here suggest a slow post-bottleneck recovery of genetic diversity (in terms of both allele numbers and heterozygosity) in the Pop2013 population in comparison to the Pop2003 population, with values from 12.800 to 13.133 and from 0.821 to 0.841 for *MNA* and *He*, respectively.

Studies have indicated that factors such as dispersive capabilities^30^,^31^,^32^,^33^,^34^ and effective population size^35^,^36^,^37^ may affect the change in genetic variation. High dispersal potential due to migration of females in most populations each year to summer calving grounds^38^,^39^ is assumed to promote frequent gene flow. Substantial gene flow was detected in our earlier investigation by examining mtDNA fragments^21^. Therefore, recovery of genetic variation via gene flow is expected, especially within the large populations. Moreover, starting in the 1990s, the Chinese government established seven Nature Reserves and constructed corridors for facilitating the migration of Tibetan antelope, both of which have likely facilitated gene flow among populations and reduced genetic loss in post-bottleneck populations.

We conclude that ample genetic diversity may still exist in the Tibetan antelope. Furthermore, the Tibetan antelope has shown a slight increase in genetic variation during the past 11-year period. In this sense, the results of the present study suggest that protection efforts did not arrive too late for the Tibetan antelope and provide molecular evidence for the effectiveness of conservation strategies.

## Additional Information

**How to cite this article**: Du, Y. *et al*. Microsatellite Loci Analysis Reveals Post-bottleneck Recovery of Genetic Diversity in the Tibetan Antelope. *Sci. Rep.* **6**, 35501; doi: 10.1038/srep35501 (2016).

## Supplementary Material

Supplementary Information

## Figures and Tables

**Table 1:** Primer pairs of 15 microsatellite loci in Tibetan antelope.

Locus	Primers	Core sequence	Ta°C
P1	F: AGCAAGCATTTGTCTGTCAGT	(AC)12(CA)14	52.8
	R: GTATGGCAGGTGGGGAAT		
P6	F: TGATCCATAAAACCAGGGGA	(GT)14	57.5
	R: GTGGAATGAGTCCATGCCTT		
P9	F: GACATTTCAATTCTTCTGCTTTAG	(CT)10(CA)14	58
	R: CAAGATGGCAGTGTCTTCTCA		
P17	F: TCTTGTGATCTCTTCCAGTAGAG	(TC)6cgctc(TC)9cacac(CA)14	54
	R: CGTCAGGCAATGAAGGTAG		
P24	F: CAGATCCCTGAAGAATGAGG	(AC)16	54.5
	R: GAGGAAGAGGATGGAGCAG		
P63	F: ATTTTCACTCCCTGCACCAA	(TA)11	59.5
	R: CTCATGGGGTAAAAGGCAGA		
P67	F: CCGGGGTGCAATTAGAGTAA	(AC)19	54.5
	R: GACTGCAATGGGTTTGTGTG		
P73	F: CACTGCCCAAGAGAACAAGA	(CT)6t(AC)12	57.5
	R: TTTTCTGGGGTGCAAGTTTC		
P75	F: GGGAAGGAGGTTCAAGAGGA	(TG)9	61
	R: CCACCATAAACTTTGTTGCCA		
P78	F: TGAATTATCCGTGTGGCAGA	(GT)7t(TG)12cgtgcgtgtgtgcatgtgtgtgtgcgtgt(GTGC)5	61.5
	R: GTCCCTCCGTGTCTGTCTGT		
P90	F: TGCAGTGGCCATCATGTAAT	(AT)14	61.5
	R: ATGTGTGCAAGTCACTTCTTTAAT		
P96	F: CAGTCATTCAAGACCAAGCG	(TC)15(CA)16	49.5
	R: CATTTTCACAAATTGAGCCCT		
P113	F: CTGACTTCTTTCTCCCTACGA	(CT)28	54
	R: CAACCACTTTTGGATTCACAG		
P154	F: CAAGGGATCATTTCAATGCT	(AGT)9	58.5
	R: GATACGACTGAGCGACTTGA		
P160	F: AAGAGGCAGCACCGTACA	(TGC)20	52.5
	R: CTATGAAAGAAAGAGCCAGAGT		

**Table 2:** Genetic diversity at microsatellite loci of Pop2003 and Pop2013 of Tibetan antelope.

Locus	Population	NC	NA	Ho	He	PIC		I
P1	Pop2003	92	11	0.804	0.776	0.750	0.774	1.875
	Pop2013	94	11	0.851	0.818	0.790		1.968
P6	Pop2003	94	8	0.660	0.636	0.561	0.623	1.240
	Pop2013	94	8	0.702	0.721	0.669		1.497
P9	Pop2003	94	14	0.830	0.840	0.818	0.827	2.165
	Pop2013	94	16	0.809	0.847	0.825		2.210
P17	Pop2003	94	13	0.745	0.775	0.746	0.744	1.883
	Pop2013	90	9	0.556*	0.772	0.738		1.750
P24	Pop2003	94	12	0.894	0.836	0.811	0.850	2.068
	Pop2013	94	13	0.957	0.892	0.872		2.310
P63	Pop2003	94	10	0.851	0.824	0.792	0.820	1.890
	Pop2013	94	13	0.809	0.867	0.843		2.170
P67	Pop2003	92	12	0.739	0.817	0.785	0.785	1.947
	Pop2013	84	11	***0.643***	0.810	0.771		1.809
P73	Pop2002	94	11	0.660*	0.856	0.832	0.850	2.102
	Pop2013	92	11	0.196*	0.866	0.841		2.129
P75	Pop2003	94	14	0.957	0.900	0.880	0.896	2.352
	Pop2013	92	15	0.870	0.920	0.903		2.524
P78	Pop2003	90	17	0.933	0.886	0.865	0.858	2.380
	Pop2013	94	16	0.809	0.863	0.840		2.248
P90	Pop2003	94	14	0.745	0.842	0.814	0.833	2.075
	Pop2013	92	12	0.804	0.870	0.845		2.159
P96	Pop2003	92	12	0.717	0.765	0.741	0.723	1.898
	Pop2013	94	12	0.766	0.726	0.697		1.728
P113	Pop2003	94	19	0.957	0.925	0.909	0.913	2.678
	Pop2013	94	21	0.936	0.920	0.904		2.719
P154	Pop2003	94	14	0.979*	0.767	0.739	0.799	1.911
	Pop2013	94	16	***0.957***	0.857	0.838		2.334
P160	Pop2003	94	11	0.830	0.867	0.841	0.851	2.101
	Pop2013	94	13	0.872	0.873	0.849		2.166
Mean	Pop2003	/	12.929	0.832	0.818	0.789		2.033
	Pop2013	/	13.286	0.810	0.840	0.813		2.114

Values of Ho marked in italic and bold indicated significant deviation (*P* < 0.05) from HWE. Population deviated from HWE after Bonferroni correction (adjusted α = 0.05/15) indicated by an asterisk. Mean values of genetic diversity parameters were calculated using 14 loci of SSR (except locus P73).

**Table 3:** Results (P-values) of bottleneck testing under three models.

Population	Test	IAM	TPM	SMM
Pop2003	Sign test: No. of loci with heterozygosity excess (probability)	7.940 (p = 0.004)	8.240 (p = 0.339)	8.300 (p = 0.065)
	Standardized differences test: T2 values (probability)	2.711 (p = 0.003)	−0.35 (p = 0.363)	−1.609 (p = 0.054)
	Wilcoxon test (Probability of heterozygote excess)	0.001	0.665	0.923
Pop2013	Sign test: No. of loci with heterozygosity excess (probability)	7.990 (p = 0.024)	8.260 (p = 0.681)	8.310 (p = 0.000)
	Standardized differences test: T2 values (probability)	2.418 (p = 0.008)	−0.934 (p = 0.175)	−2.343 (p = 0.010)
	Wilcoxon test (Probability of heterozygote excess)	0.000	0.805	0.985

Analyses with BOTTLENECK used three microsatellite mutation models: infinite allele model (IAM), two-phase model (TPM) and stepwise mutation model (SMM).

**Table 4:** Estimation of theta (*θ*) in Pop2003 and Pop2013 of Tibetan antelope.

Locus	Pop2003	Pop2013	Mean	s.d.
P1	3.467	4.484	3.976	0.719
P6	1.744	2.586	2.165	0.595
P9	5.262	5.553	5.408	0.206
P17	3.438	3.387	3.412	0.036
P24	5.088	8.261	6.674	2.244
P63	4.691	6.536	5.614	1.304
P67	4.479	4.250	4.365	0.162
P75	8.957	11.533	10.245	1.822
P78	7.783	6.285	7.034	1.059
P90	5.344	6.681	6.012	0.945
P96	3.250	2.649	2.949	0.425
P113	12.326	11.489	11.907	0.592
P154	3.298	5.971	4.635	1.890
P160	6.497	6.847	6.672	0.247
Mean	4.503	5.235	4.869	0.518

**Table 5:** Summary of genetic diversity parameters of microsatellite data of several Bovidae species.

Species	Researcher	MNA	He
Banteng *Bos javanicus*	40	2.42	0.47
Wild gaur *B. gaurus*	41	2.2	0.091–0.835
Yak *B. grunniens*	42	11.69	0.616
Przewalski’s gazelle *Procapra przewalskii*	43	5.98	0.780
	44	5.85	0.552
American Bison *Bison bison*	45	3.56–5.00	0.522–0.652
Dorcas gazelle *Gazella Dorcas*	46	2.2–7.0	0.466–0.727
African wild ass *Equus africanus*	47	5.06	0.59
Huemul *Hippocamelus bisulcus*	48	5.25	0.461
Chiru *Pantholops hodgsoni* Pop2003/Pop2013	20	9.4	0.838
	This study	12.800/13.133	0.821/0.841
