# Supplementary material for: Microsatellite Loci Analysis Reveals Post-bottleneck Recovery of Genetic Diversity in the Tibetan Antelope
Source: Sci Rep. 2016 Oct 14;6:35501. doi: 10.1038/srep35501 (PMC5064351; doi:10.1038/srep35501)
Supplement: Supplementary Information [file srep35501-s1.doc]

**Title page**

**Microsatellite Loci Analysis Reveals Post-bottleneck Recovery of Genetic Diversity in the Tibetan Antelope**

Yurong Du^1^, Xiaoyan Zou^2^, Yongtao Xu^3^, Xinyi Guo^3^, Shuang Li^1^, Xuze Zhang^4^, Mengyu Su^1^, Jianbin Ma^1*^, Songchang Guo^2*^

**Supplementary Information**

Table S1 Test of the linkage disequilibrium on each locus for Pop2003 and Pop2013

|  | P1 | P6 | P9 | P17 | P24 | P63 | P67 | P75 | P78 | P90 | P96 | P113 | P154 | P160 |
| --- | --- | --- | --- | --- | --- | --- | --- | --- | --- | --- | --- | --- | --- | --- |
| P1 |  | 0.6531 | **0.0096** | 0.5881 | 0.6689 | 0.2315 | 0.2298 | 0.3661 | 0.4734 | 0.0749 | 0.9808 | 0.3883 | 0.3678 | 0.1546 |
| P6 | 0.1891 |  | 0.9943 | 0.4277 | 0.3695 | 0.3447 | 0.8267 | 0.7885 | 0.3299 | 0.4018 | 0.0762 | 0.5435 | 0.2395 | 0.3916 |
| P9 | **0.0161** | **0.0178** |  | 0.8015 | 0.4531 | 0.9648 | 0.2637 | 1.0000 | **0.0175** | 0.2064 | 0.7179 | 1.0000 | 0.8679 | 0.4356 |
| P17 | 0.1284 | 0.1354 | 0.1491 |  | 0.9440 | 0.6799 | 0.9794 | 0.8294 | 0.4363 | 0.9596 | 0.8298 | 0.4315 | 0.4372 | 0.7228 |
| P24 | **0.0005*** | 0.5627 | **0.0186** | **0.0123** |  | 0.4552 | 0.8253 | 0.5853 | 0.2325 | 1.0000 | **0.0102** | 1.0000 | 0.0568 | 0.7508 |
| P63 | 0.2532 | 0.0920 | 0.0970 | 0.0595 | **0.0438** |  | 0.9793 | 0.4845 | 0.8407 | 0.7969 | 0.1444 | 0.4322 | 0.3008 | 0.9377 |
| P67 | **0.0065*** | 0.1822 | 0.3519 | 0.2559 | **0.0227** | **0.0017*** |  | 1.0000 | 0.3189 | 0.8665 | 0.9401 | 1.0000 | 0.8328 | 0.0514 |
| P75 | **0.0138** | **0.0061** | **0.0245** | **0.0007*** | **0.0006*** | **0.0008*** | **0.0029*** |  | 0.4505 | 1.0000 | 0.6689 | 1.0000 | 0.1357 | 0.5788 |
| P78 | **0.0097** | 0.4827 | **0.0069** | 0.1026 | **0.0015*** | 0.0735 | 0.0592 | **0.0001*** |  | 0.6249 | 0.7051 | 1.0000 | 0.9732 | 0.6047 |
| P90 | 0.0790 | 0.2333 | **0.0119** | 0.5307 | 0.2308 | 0.0533 | 0.1025 | 0.0740 | 0.0878 |  | 0.1988 | 1.0000 | 0.8828 | 0.1486 |
| P96 | **0.0152** | 0.7580 | **0.0431** | 0.1512 | **0.0019*** | **0.0046** | 0.5054 | **0.0389** | **0.0383** | **0.0378** |  | 1.0000 | 0.0119 | 0.4707 |
| P113 | **0.0001*** | **0.0352** | **0.0044** | **0.0051** | **0.0000*** | **0.0120** | **0.0020*** | **0.0000*** | **0.0030*** | **0.0006*** | **0.0099** |  | 1.0000 | 0.2434 |
| P154 | **0.0061** | 0.1419 | **0.0271** | 0.0662 | **0.0099** | **0.0000*** | **0.0163** | **0.0013*** | 0.0947 | 0.2732 | **0.0264** | **0.0016*** |  | 0.7976 |
| P160 | **0.0043** | **0.0043** | **0.0006*** | 0.1108 | **0.0015*** | **0.0104** | 0.4349 | **0.0123** | **0.0033*** | 0.2745 | 0.2783 | **0.0016*** | **0.0161** |  |

Bold values indicate significant departures (*P*<0.05) from Linkage disequilibrium.

* denotes significant departure from Linkage disequilibrium after Bonferroni correction (adjusted α = 0.05/14).
